# Bilateral Vision Loss Secondary to Pachymeningitis in a Patient with IgG4-Related Disease

**DOI:** 10.3389/fneur.2014.00192

**Published:** 2014-10-13

**Authors:** Lucas Ramirez, Andrea D’Auria, Adeel Popalzai, Nerses Sanossian

**Affiliations:** ^1^Roxanna Todd Hodges Comprehensive Stroke Clinic, University of Southern California, Los Angeles, CA, USA

**Keywords:** IgG4-related disease, pachymeningitis, vision loss, IgG4, dural thickening

## Abstract

IgG4-related disease (IgG4-RD) is a recently recognized fibroinflammatory condition associated with disease in nearly every organ, including the meninges. A proportion of idiopathic hypertrophic pachymeningitis cases may involve a component of meningeal IgG4-RD. We present a patient with severe bilateral vision loss found to have thickening of the dura mater on MRI, and subsequently diagnosed with IgG4-RD after dural biopsy.

## Case

A 44-year-old man presented to the emergency department with progressive vision loss. He reported worsening vision for 8 days to near blindness, new-onset severe, and pressure like, non-pulsatile headache. Past medical history was remarkable for diabetes, hypertension, dyslipidemia, and rheumatoid arthritis. Vital signs were stable and the neurological examination showed no light perception on the OS, and only hand motion perception on the OD.

**Figure 1:**
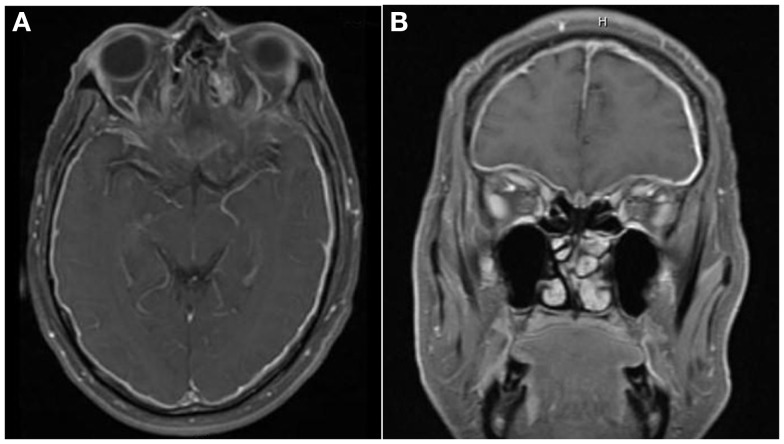
**There is diffuse smooth enhancement of the pachymeninges (A), left greater than right, with apparent thickening (B)**. Globes are normal in their size, shape, and signal intensity on all pulse sequences. No evidence of intra or extraconal soft tissue mass. Bilateral enhancement of posterior intraconal optic nerve sheath with normal signal intensity of optic nerves.

Non-contrast CT of the head showed no intracranial abnormality. Ophthalmological evaluation found no apparent explanation for the visual loss. Dexamethasone therapy was initiated. Contrast-enhanced MRI of the brain done 1 day later showed diffuse thickening and enhancement of the dura, greater on the left side (Figure 1A). MRI of the orbits showed bilateral enhancement of the posterior aspect of the intraconal optic nerve sheath (Figure 1B). Lumbar puncture demonstrated an opening pressure of 32 mm H_2_O, glucose 90 mg/dL, protein content of 44 mg/dL, RBC 1, and WBC 21 (80% lymphocytes, 16% monocytes, 4% neutrophils). Gram stain, cultures, and cytology were negative. IgG index was 1.09 (reference <0.66), and IgG was 13.3 mg/dL (range 0.8–7.7). A left frontal dural biopsy was performed.

**Figure 2:**
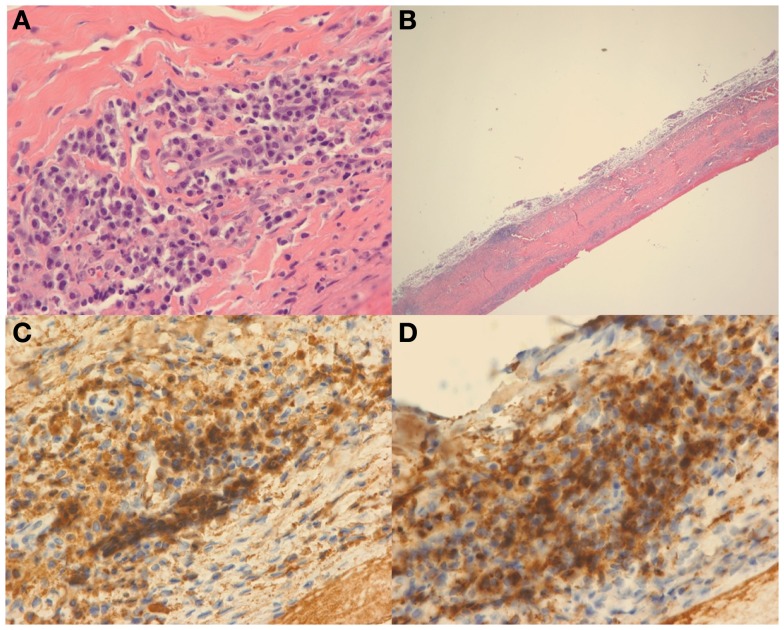
**Hematoxylin and eosin (H&E) stain at high power (A) and low power (B) demonstrate dural thickening with prominent lymphoplasmacytic infiltrate**. On high power **(A)**, the infiltrate is composed of mostly plasma cells. Immunohistochemical stain for IgG **(C)** and IgG4 **(D)** demonstrate that most of the plasma cells are positive for IgG and that the vast majority of the plasma cells are IgG4 positive.

The patient’s vision continued to improve with steroid therapy and on day 10, VA was noted to be 20/20 on the OD and 20/30 on OS. Pathological evaluation showed pachymeningitis with increased IgG4 plasma cells (Figure 2). Given the clinical, laboratory, and pathology results, a diagnosis of IgG4-related disease (IgG4-RD) was made. Serum IgG4 drawn on the day of hospital discharge revealed elevated levels of 182.0 (reference range 4–86).

## Discussion

IgG4-related disease is a recently recognized fibroinflammatory condition first linked to autoimmune pancreatitis (AIP) in 2001 when pancreatic specimens from patients with AIP were found to have large numbers of IgG4 positive cells (1). In 2003, Kamisawa et al. reported extrapancreatic lesions in patients with AIP containing histopathology identical to that found in the pancreas. It was thus suggested that AIP was part of a larger systemic disease and not just confined to the pancreas (2).

Since then, IgG4-RD has been associated with disease involving nearly every organ system including pancreas, salivary glands, lacrimal glands, thyroid, pituitary, lungs, aorta, pericardium, liver, biliary tree, kidneys, retroperitoneum, breast, prostate, lymph nodes, skin, periorbital tissues, and the meninges (1–3). Tissues from involved organs demonstrate similar histopathological features of lymphoplasmacytic infiltration, many IgG4 positive plasma cells, and fibrosis (1, 2).

The meninges may be involved either intracranially or intraspinally with a predilection for involvement of the pachymeninges rather than the leptomeninges (1). The MRI findings of thickened dura, along with characteristic histology of fibrosis and lymphoplasmacytic infiltrates, fulfill the clinicopathological features of hypertrophic pachymeningitis (HP), a rare fibroinflammatory lesion causing thickening of cranial and/or spinal dura (4). HP may be due to infections such as tuberculosis and syphilis, systemic autoimmune diseases, neoplasms, or may be idiopathic (4). It is believed that a proportion of previously reported cases of idiopathic hypertrophic pachymeningitis (IHP) actually represent IgG-RD. A recent retrospective study analyzed 10 cases of IHP and showed evidence of IgG4-RD histopathology in 5 of the 10 (2, 3).

IgG4-related vision loss is rare. IgG4-RD can present as non-specific constitutional symptoms at the time of diagnosis, or more likely as a sub-acute expanding mass, with disease confined to a single organ for many years (1, 2). A proportion of cases of IHP have demonstrated IgG4-RD. IHP presents with radiculomyelopathy, headaches, cranial nerve palsies, papilledema, and unilateral or bilateral visual loss (1, 2, 4, 5). These presentations can also be included as those of IgG4-RD. The visual loss associated with HP has been attributed to inflammatory optic neuropathy. Other potential mechanisms of visual loss include infiltrative and compressive retrobulbar optic neuropathy (6, 7).

## Conclusion

We describe a patient with visual loss due to pachymeningitis from IgG4-RD. We believe that a proportion of IHP actually represents IgG4-RD and that clinicians should consider testing for this condition with biopsy and IgG4 immunostaining. Establishing a diagnosis of IgG4-RD is important in planning immunosuppressive therapy to prevent future complications.

## Teaching Points

Consider IgG4-RD in the differential diagnosis of HP. Definitive diagnosis of IgG4-RD requires biopsy demonstrating characteristic histopathology along with IgG4 immunostaining. Initial outcome is favorable with corticosteroids, though response is often unsustained in the long-term, thus, additional immunosuppressive agents may be required. Patients with single organ involvement of IgG4-RD should be followed and screened for systemic involvement as disease may develop in multiple locations.

## Conflict of Interest Statement

The authors declare that the research was conducted in the absence of any commercial or financial relationships that could be construed as a potential conflict of interest.

## References

[1] Stone JH Zen Y Deshpande V IgG4-related disease. N Engl J Med (2012) 366(6):539–51 10.1056/NEJMra1104650 22316447

[2] Khosroshahi A Stone JH A clinical overview of IgG4-related systemic disease. Curr Opin Rheumatol (2011) 23(1):57–66 10.1097/BOR.0b013e3283418057 21124086

[3] Lindstrom KM Cousar JB Lopes MB IgG4-related meningeal disease: clinico-pathological features and proposal for diagnostic criteria. Acta Neuropathol (2010) 120(6):765–76 10.1007/s00401-010-0746-2 20844883

[4] Chan SK Cheuk W Chan KT Chan JK IgG4-related sclerosing pachymeningitis: a previously unrecognized form of central nervous system involvement in IgG4-related sclerosing disease. Am J Surg Pathol (2009) 33(8):1249–52 10.1097/PAS.0b013e3181abdfc2 19561447

[5] Lam BL Barrett DA Glaser JS Schatz NJ Brown HH Visual loss from idiopathic intracranial pachymeningitis. Neurology (1994) 44(4):694–8 10.1212/WNL.44.4.694 8164828

[6] Chan JW Acute monocular visual loss in carcinomatous hypertrophic pachymeningitis mimicking giant cell arteritis. J Clin Rheumatol (2006) 12(1):30–1 10.1097/01.rhu.0000200423.12270.18 16484877

[7] Liewluck T Schatz NJ Potter PF Romaguera RL Compressive retrobulbar optic neuropathy due to hypertrophic pachymeningitis. Intern Med (2008) 47(19):1761–2 10.2169/internalmedicine.47.1412 18827435

